# Digital pair-matching of iliac bones: pilot study on a three-dimensional approach with models acquired through stereophotogrammetry

**DOI:** 10.1007/s00414-022-02895-x

**Published:** 2022-10-05

**Authors:** Andrea Palamenghi, Debora Mazzarelli, Annalisa Cappella, Danilo De Angelis, Chiarella Sforza, Cristina Cattaneo, Daniele Gibelli

**Affiliations:** 1 Laboratorio di Antropologia e Odontologia Forense (LABANOF), Sezione di Medicina Legale, Dipartimento di Scienze Biomediche per la Salute, Università degli Studi di Milano, Via L. Mangiagalli 37, 20133 Milan, Italy; 2 Laboratorio di Anatomia Funzionale dell’Apparato Stomatognatico (LAFAS), Dipartimento di Scienze Biomediche per la Salute, Università degli Studi di Milano, Via L. Mangiagalli 31, 20133 Milan, Italy; 3 Dipartimento di Scienze Biomediche per la Salute, Università degli Studi di Milano, Via Luigi Mangiagalli 31, Milan, Italy; 4 U.O, Laboratorio di MorfologiaUmanaApplicata, IRCCS Policlinico San Donato, San Donato Milanese, Italy

**Keywords:** Commingled remains, Pair-matching, Stereophotogrammetry, 3D-3D superimposition, Pelvic bones

## Abstract

Three-dimensional (3D) pair-matching has brought about an innovative approach for the analysis of commingled skeletal remains, and it has been tested on bone models acquired through CT and laser scans. Here, 3D models of 40 innominate bones (20 left and 20 right) of 20 documented male individuals from a cemeterial skeletal collection were acquired through a stereophotogrammetric device (VECTRA M3, Canfield Scientific, Inc.). The ventral iliac surface was chosen as the anatomical region of interest (ROI) for the analysis. Each left ROI was mirrored and superimposed on the matching right ROI (contralateral element from the same individual) and mismatching ROIs (contralateral elements from different individuals). The point-to-point distance between models was calculated through the Vectra Analysis Module (VAM) software and the root mean square (RMS) point-to-point distance value was used to evaluate the sorting performance of the method, in terms of sensitivity and specificity rates. Differences in RMS between matches and mismatches were investigated through a Student’s *t* test (*p* < 0.05). The state of preservation of the remains was assessed following an index of anatomical completeness and differences in RMS distances of true matches according to different anatomical completeness were assessed through the Mann–Whitney test (*p* < 0.05). RMS point-to-point distances of matches and mismatches were significantly different (*p* < 0.01), being the matches lower than mismatches. The RMS threshold of 2.9 mm identified all the true pairs; the test was 100% sensitive and 51% specific. The RMS of matches with a better state of preservation are significantly lower than the less preserved matches (*p* < 0.05). In general, a low RMS distance value may indicate a true match, being it to be further verified. The 3D approach for sorting innominate bones provides a valid screening test that could complete subjective and osteometric methods with numerical evidence of the match. Preliminary data suggest a possible relation between RMS distance values and taphonomic condition, which would benefit from further research.

## Introduction

Forensic anthropologists have been extensively deployed to recover and identify commingled skeletal remains, both from forensic and archaeological contexts. They took an active part in forensic humanitarian task forces in mass disasters [1, 2] and in investigations of mass burials from genocides [3–5]. In the archaeological field, ossuaries—such as the ones of Indian groups in North America [6] or the sepulchre of Ospedale Maggiore of Milan, with its nine underground chambers filled to capacity with skeletal remains from different time periods of Italian history [7]—are frequent secondary deposits presenting a mixing of individuals.

Human commingled skeletal remains result in a highly intricate scenario for the process of identification, as the forensic anthropologist must sort the bones and ‘rebuild’ the single individual. L’Abbè [8] described it as “a daunting task”, especially when origins and number of individuals involved are unknown. This aspect slows down the analysis yet sorting the remains to their persons-of-origin is crucial before they can be properly identified [9, 10]. One of the first steps of the sorting procedures is the pair-matching of bilateral elements. Traditional approaches include morphological and visual evaluations (visual pair-matching), which rely mainly on morphological examination and on the experience of the observer [6]. Since the early 2000s, the literature has focused on the challenges that come with commingled remains, aiming to devise systematic methods to analyse and efficiently segregate the individuals [6, 10]. Osteometric statistical methods were developed to overcome the limitations of traditional subjective methods, but they are still restrained from some limitations, such as the size of the commingled assemblage and the disparity of size among the individuals in the sample [10, 11]. Furthermore, osteometric analyses rely on the identification of anatomical landmarks, which can lead to the introduction of possible error by the observers. Moreover, they strongly depend on reference populations, therefore the application to unknown samples is limited [12].

In an attempt to overcome these shortcomings, Karell et al. [12] devised a novel three-dimensional (3D) method for the pair-matching of right and left humeri. Both manual and automated versions of the method were proposed. The mesh-to-mesh value comparison (MVC) method superimposes 3D models of bones and calculates the point-to-point distance in millimetres between the two meshes, which is used to discern pair-matches and mismatches. The assumption is that a low mesh-to-mesh value indicates the provenance from the same individual. According to the authors, the MVC method does not depend on sex, chronology, or reference population of the specimens. This has been tested on 3D models of temporal bones [13], phalanges [14], and clavicles [15], as well. The models derived from computerized tomography (CT) scans or from surface scans of bare bones. Literature currently lacks information on the possible use of other acquisition techniques and of other bones for the digital sorting of mixed bones. Within this perspective, this pilot study investigates the 3D-3D superimposition method for the pair-matching of iliac bones that were acquired through a different tool, that is, stereophotogrammetry. In the analysis of surfaces, it has been demonstrated that photogrammetry is an accurate alternative to more expensive acquisition systems (such as CT scans) as high-resolution models that produce reliable results can be obtained through this mean [39]. Stereophotogrammetry, particularly the device used in this study (VECTRA-3D® M3: Canfield Scientific, Inc., Fairfield, NJ), is among the gold standard techniques for 3D surfaces acquisition [40, 41]; moreover, its ability to automatically re-build the geometry of the surface is beneficial time-wise. The affiliated laboratory is equipped with the above-mentioned stereophotogrammetry machine, which has been successfully used in recent publications to acquire bone surfaces [26]. It thus represents a valuable tool for the reproduction of bone models which is worth exploring within the field of virtual anthropology.

The bones were selected from a documented skeletal collection and have a similar and well-known taphonomic profile (15 years of burial). The aim of the work is twofold: to investigate the pair-matching of iliac bones through the superimposition of virtual bone models acquired with stereophotogrammetry and to introduce the possible shortcomings of the technique when applied to bones with a suboptimal state of preservation.

## Materials and methods

Forty innominate bones (20 left and 20 right) from 20 documented individuals were selected from *Collezione Antropologica LABANOF* (CAL), a modern skeletal collection of individual skeletons, which is hosted at the *Laboratorio di Antropologia e Odontologia Forense* (LABANOF) of the University of Milan, Italy [16]. The article 43 of the Presidential Decree of the Italian Republic (DPR) n° 285 (September 10th, 1990) grants the universities the right to collect unclaimed skeletal remains for educational and research purposes. The individuals included in this study are adult males, aged between 29 and 86 years (mean age: 52.6 ± 18 years), without pathological signs on the hip bones. At the time of death, the bodies were buried inside individual coffins in cemeteries across the city of Milan and were exhumed after 15 years. When the individuals were completely skeletonized, the remains were placed inside zinc boxes and stored at the laboratory facilities.

**Fig. 1:**
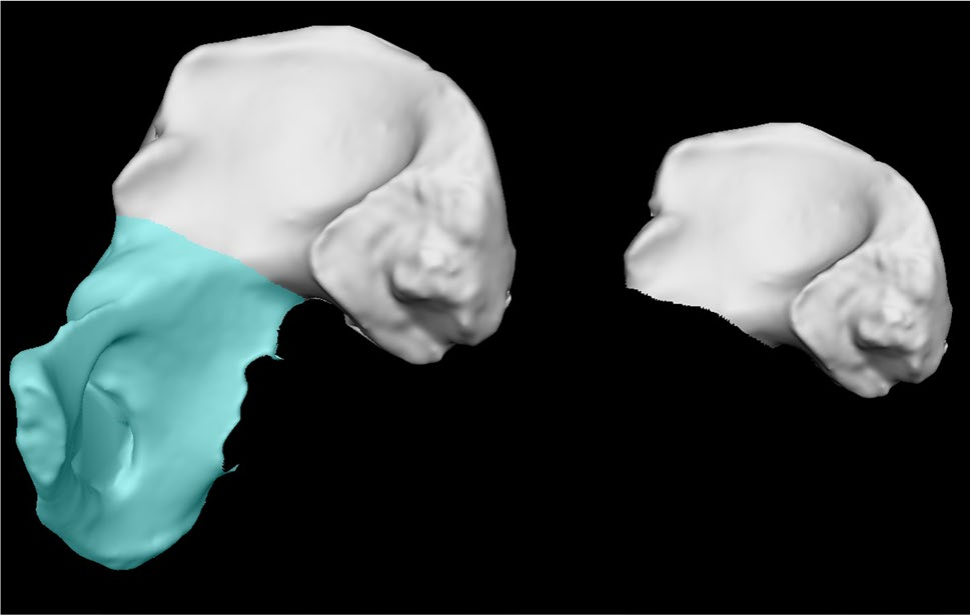
Isolation of the ventral iliac surface as region of interest (ROI). A quasi-straight line was drawn from the inferior margin of the anterior-inferior iliac spine to the most superior point of the greater sciatic notch with the editing tool on VAM. The blue region of the surface (on the left) was selected and then deleted to isolate the anterior surface of the ilium (on the right)

The rationale for choosing the innominate bone is that it is highly informative for sex [17] and age estimation [18, 19] of unknown skeletal remains; thus, the correct sorting of this skeletal district may provide crucial insights to the investigation. Moreover, it has a planar structure that can be easily acquired through stereophotogrammetry. The virtual models of the bones were acquired with the stereophotogrammetric device VECTRA M3 (Canfield Scientific, Inc., Fairfield, NJ), which captures three pictures from different angles and automatically rebuilds the virtual surface of the object. Technical characteristics of VECTRA-3D® M3 include sample density: 1.2 mm geometric resolution; capture volume: 400 × 300 × 250 mm; and speed of acquisition: 3.5 ms [40]. Since some bones did not present considerable portions (i.e., pubis and ischium) due to postmortem damage, the ventral iliac surface was chosen as the region of interest (ROI) and isolated with the editing tool of the software Vectra Analysis Module (VAM, Canfield Scientific, Inc., Fairfield, NJ). The ROI included the whole ventral surface of the ilium which was limited inferiorly by a quasi-straight line that goes from the inferior margin of the anterior-inferior iliac spine and the most superior point of the greater ischiatic notch (Fig. 1).

**Fig. 2:**
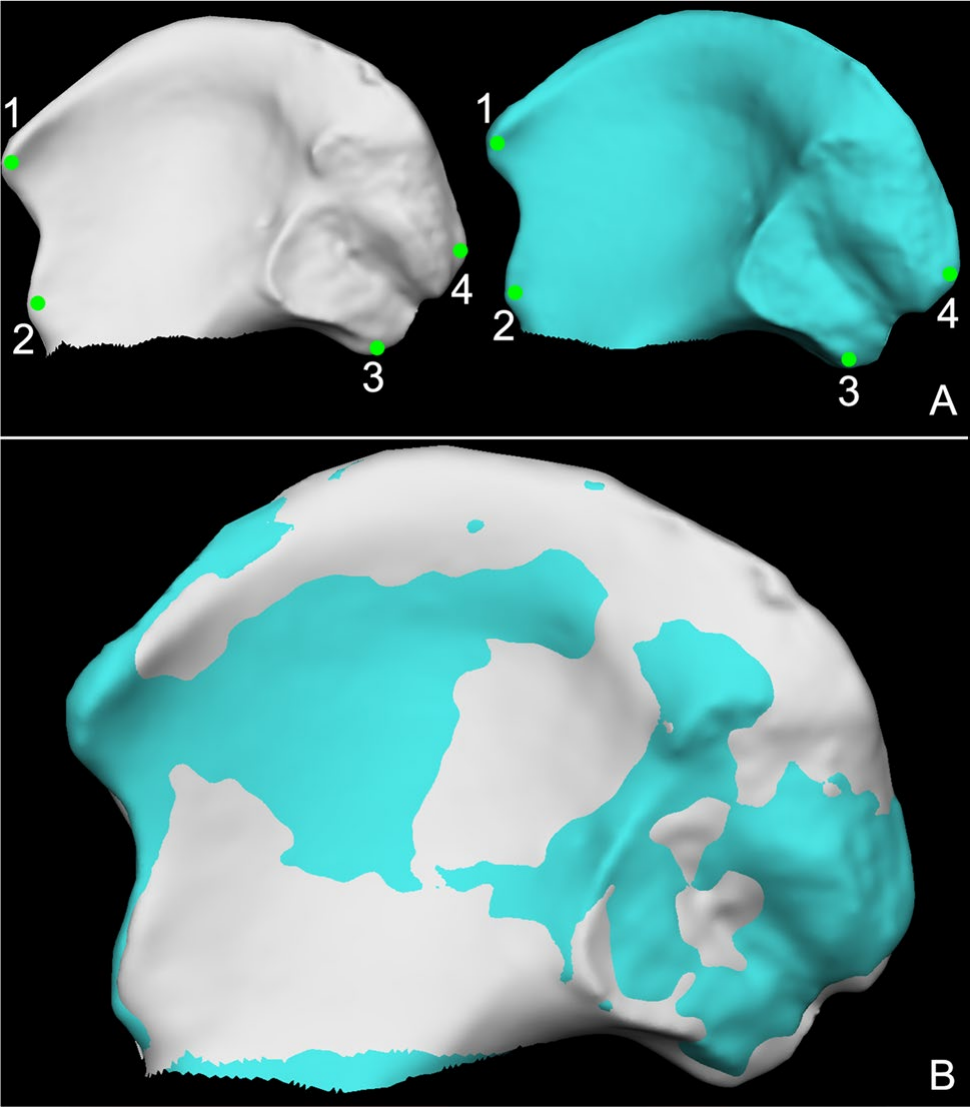
Registration protocol of the iliac surfaces. **A** Corresponding anatomical landmarks (green dots) placed to near the two models before manual registration. In grey colour is the right iliac surface, whereas in light blue colour is the mirrored left iliac surface. The landmarks were sequentially placed on the anterior superior iliac spine (1), anterior inferior iliac spine (2), posterior inferior iliac spine (3), and posterior superior iliac spine (4). **B** Result of the automatic registration of the two iliac surfaces performed by the software (the left iliac surface is showed in blue)

**Fig. 3:**
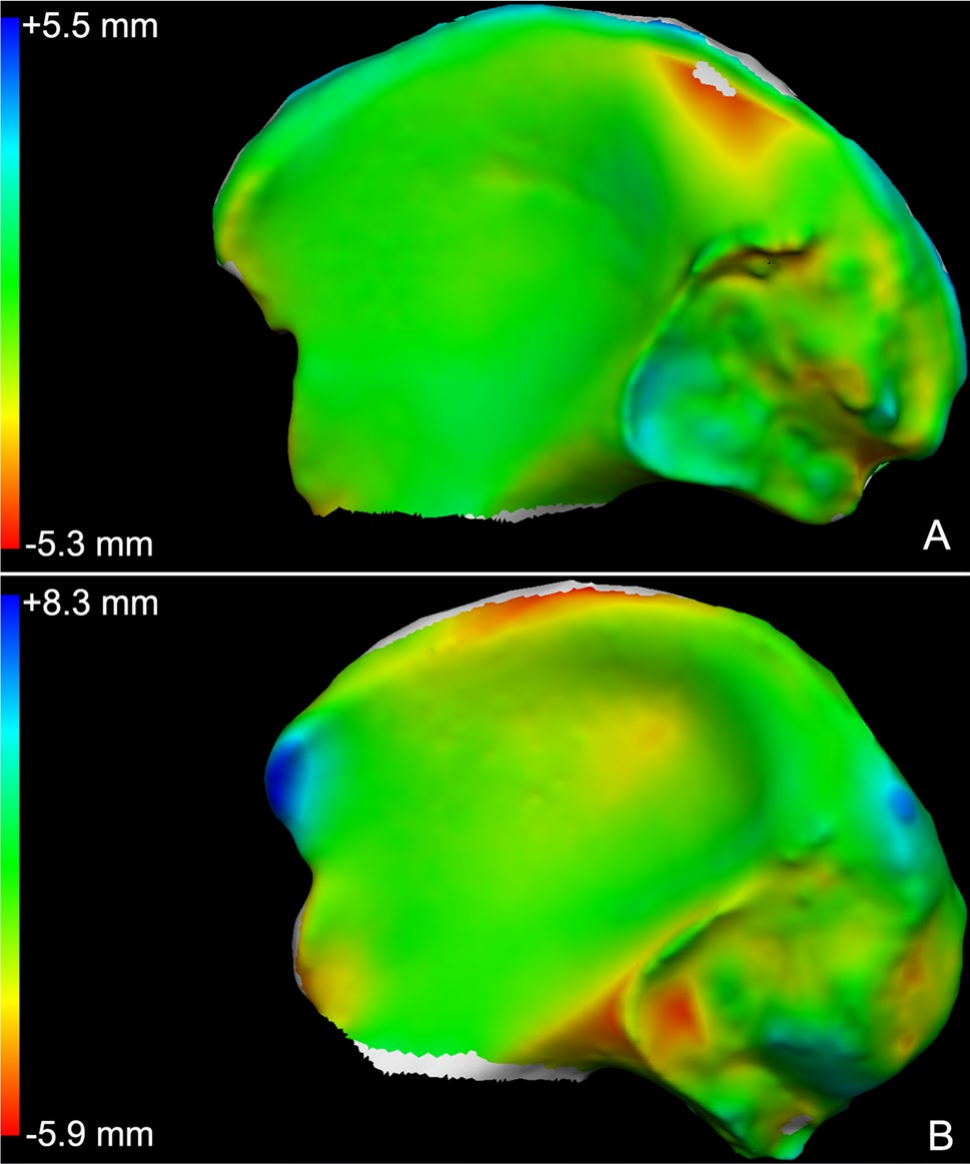
Chromatic map of the point-to-point distance between the two models. The green areas indicate coincident points on the models. In blue are the recessing areas of the left iliac surface according to the right iliac surface. In red, orange and yellow are the projecting areas of the left iliac surface according to the right iliac surface. Grey indicates the areas where the software could not identify common points between models. **A** Visualization of the point-to-point distance between the right and left iliac bones from the same individual (true pair-match). **B** Visualization of the point-to-point distance between the right and left iliac bones from two different individuals (true pair-mismatch)

A recent study pointed out that the superimposition protocol significantly influences the results of the point-to-point distance calculation between 3D surfaces [20]. Therefore, a specific superimposition protocol was devised on the VAM software. The right iliac surface was chosen as the reference model onto which the left iliac surface would be moved. After mirroring the left iliac surface, four corresponding landmarks (anterior–superior and anterior-inferior iliac spine; posterior-superior and posterior-inferior iliac spine) were placed on both surfaces by the observers (Fig. 2A). Then each mirrored left surface was superimposed on each right surface: the first step included the manual registration according to the four anatomical landmarks to near the two models; the second step was the automatic registration on the whole surface of the models, which was performed by the software according to the proprietary algorithms (Fig. 2B). The root mean square (RMS) point-to-point distance (in millimetres) of the left model to the right one was then calculated with VAM, which allows to visualize the distance between models as a chromatic map (Fig. 3). 400 point-to-point distance analyses were performed, resulting from the superimposition of the 20 known true pair-matches and the 380 true pair-mismatches.

Since the true pair-matches were known to the observers, the RMS distance values output by VAM were used as proxy to discern pair-matches and mismatches. The sorting ability of the method was assessed with sensitivity and specificity rates, by choosing a threshold equal to or under which the pair would be considered a match. Sensitivity is the proportion of true pair-matches correctly identified as such, whereas specificity indicates the proportion of true pair-mismatches correctly identified by the test. The pilot study was designed to include only males in order to avoid the possible effect of size difference between sexes: pooling specimens from males and females would possibly affect the outcome of the RMS distance values calculation by creating higher RMS distance values in the mismatch groups, thus biasing the accuracy.

Two operators with experience in 3D analysis performed the editing and superimposition of the models. Repeatability of the whole procedure was investigated through inter and intraobserver error, both expressed as absolute technical error of measurement (TEM) and relative technical error of measurement (rTEM). Differences between RMS distances of matches and mismatches were evaluated through a Student’s *t* test (*p* < 0.05). The ROI of some specimens presented postmortem damage that affected the anatomical completeness. The taphonomic condition was therefore assessed and quantified following the Anatomical Preservation Index (API) by Bello et al. [21]. All bones belonged to classes 5 (75–99% of bone preserved) and 6 (100% of bone preserved) of the scoring system. A preliminary investigation on the possible relation between RMS distance values of the known matches and the mean value of the scores of preservation assigned to the true pair was carried out through Mann–Whitney test (*p* < 0.05).

## Results

**Fig. 4:**
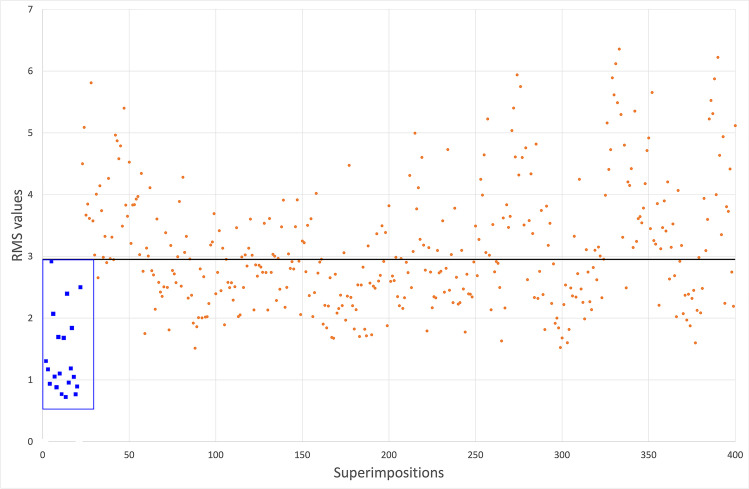
Scatter plot of the RMS point-to-point distance values from the 400 superimpositions. The x-axis is the number of the superimpositions performed. The y-axis is the RMS distance values (millimiters). Blue squares within the blue rectangle are the RMS distances of true matches. The orange dots are the RMS distance values of true mismatches. The black horizontal line marks the 2.9 mm threshold

The software VAM output 400 RMS distance values, 20 of which corresponded to the 20 true pair-matches and 380 to the known pair-mismatches (Fig. 4). RMS distance values of matches ranged between 0.73 and 2.92 mm (mean: 1.33 mm ± 0.59 mm), whereas those of mismatches were between 1.51 and 6.35 mm (mean: 3.3 mm ± 0.99 mm). The Student’s *t* test revealed that RMS distance values were different between matches and mismatches, being the matches significantly lower than mismatches (*p* < 0.01). The procedure proved highly repeatable (Table 1). Intra-observer and inter-observer relative technical error of measurement (rTEM) was 1.95% and 1.62% for matches and 1.08% and 1.21% for mismatches, respectively. Both inter-observer and intra-observer agreement could be classified as “very good” according to Camison et al. [22].

**Table 1:** Intra- and inter-observer error in matches and mismatches. *TEM*, absolute technical error of measurement; *rTEM*, relative technical error of measurement

Repeatibility	Intra TEM (rTEM)	Inter TEM (rTEM)	Agreement category (Camison et al., 2018)
Matches	0.02 mm (1.95%)	0.02 mm (1.62%)	Very good
Mismatches	0.03 mm (1.08%)	0.03 mm (1.21%)	Very good

Table 2 shows the performance of the method when the threshold was set at 2.9 mm: the method was 100% sensitive and correctly identified the 20 true pair-matches, although it generated 185 false positives which reduced the specificity rate. As for the taphonomic evaluations, 12 true pairs were assigned the API 6, meaning that they were 100% anatomically complete, whereas 8 true pairs were given the API 5, due to their suboptimal state of preservation. The Mann–Whitney test revealed that the RMS point-to-point distances of the true pairs that are anatomically complete (API = 6) are significantly lower (*p* < 0.05) than the true pairs that present heavier signs of postmortem damage.

**Table 2:** Sorting performance in terms of sensitivity and specificity of the method according to the chosen threshold

	True positive	False positive	True negative	False negative	Sensitivity	Specificity
2.9 mm threshold	20	185	195	0	100%	51%

## Discussion

The resolution of commingled assemblages may be one of the most demanding tasks for forensic anthropologists. 3D approaches have recently brought about some tools that may support traditional visual and osteometric methods and may overcome some limitations, such as the subjectivity of the observations and the dependence on reference populations. This pilot study applied the 3D-3D superimposition for digital pair-matching (originally engineered by Karell et al. [12]) to iliac bones, using virtual models of the ventral iliac surface acquired through a stereophotogrammetric device. Previous studies tested the 3D unmingling method on humeri [12] and temporal bones [13]. Other bones considered were phalanges [14] and clavicles [15], although the results were only related to the automatic version of the method. In a forensic anthropological investigation of unknown remains, the innominate bone may be a significant element to reassociate, as it is highly informative of two parameters of the biological profile, i.e., sex [17] and skeletal age at death [18, 19]. This entails that, in a commingled context, a successful sorting of this bone could be a turning point both for the biographical information that this skeletal element bears and for the re-association of other articulating bones, such as the vertebral column and femora [11, 23, 38].

Per the results of this pilot study, the method proved highly sensitive (100%), although the specificity is quite low (51%). By choosing 2.9 mm as the cut-off value to determine pair-matches, the success rate decreased in specificity, compared to the 3D pair-matching of humeri [12]. However, it is to be pointed out that the results can only be partially compared. In the original study, the reported sensitivity and specificity rates were both 100% for the manual superimposition, but the performance was evaluated based on the lowest common value criterion [12]. According to this criterion, the three lowest values for each pair were assessed, and the pairs were considered a match when the left on right and the right on left mesh-to-mesh values agreed. This is a relative criterion [9] and could be an ineffective mean to assess the pairs because the true match value may not fall within the first three lowest values, especially if larger samples are considered. Karell et al. [12] mentioned that when the cut-off value of 1.035 mm was considered, 16 additional values—that actually represented mismatches—were included in the positive group. The abstract presented for the digital pair-matching of temporal bones did not report the performance of the method based on a threshold [13]. If, on one side, the 2.9 mm threshold produced unbalanced sensitivity and specificity rates in this pilot study, on the other side it allowed to identify all the true matches, so that no false negative was generated. In a possible forensic scenario, including all matches in the true positive group is essential for a successful identification of the true pairs. The choice of a more specific threshold that would decrease the number of false positive may lead to discounting true matches that present higher RMS distance value. Besides, this study considered the best-case scenario of a closed system, where all bones are recovered and paired. Next studies will consider worse-case scenario, in order to fully understand whether this circumstance could introduce limitations to the virtual sorting of iliac bones. This pilot study included only males that have reached skeletal maturity, so to create a homogenous sample that is not biased by differences in size entailed with age and between sexes. The authors argue that pooling together ilia of different sexes may influence the reliability of the method, as the outcomes of distance calculation would include higher RMS distance values. Although a recent application of the automatic version of 3D-3D superimposition for the pair-matching of clavicles concluded that age and pathology do not significantly affect the process in a small subset [15], this preliminary evidence is worth further investigation, since dysmorphism between right and left bones of the same individual due to pathological processes is likely to produce unreliable models that would be possibly deemed as mismatches by the technique.

Thus far, from a practical standpoint, it can be reasoned that the RMS distance values can provide indications, rather than a sound confirmation of the match or mismatch. Specifically, a high RMS distance value most likely indicates that the pair can be discounted, whereas a low RMS distance value arguably belongs to a true pair-match. However, given the considerable number of false positives that were generated with this threshold, the correct sorting of the pair indicated by the low RMS distance should be verified with further analyses. Nevertheless, the method could be used as a screening test for a first step of narrowing down the pairs that would be further examined to ascertain the match. Another major difference with the literature is the acquisition mean: the 3D models of this study were acquired through a stereophotogrammetric device, whereas previous studies employed models from CT and surface scans [12–15]. Photogrammetry is expanding in the biological anthropology field [24]. The affiliated laboratory LAFAS is equipped with the stereophotogrammetric device Vectra M3 (Canfield Scientific, Inc.), whose ability to accurately reproduce the virtual model of the object has been validated [25]. Although the reproduction of meshes from bare bones is still to be extensively explored, this device has been recently employed to acquire models for the re-association of articulating bones [26]. Nonetheless, such device was suitable for the acquisition of the planar structure of the innominate bone. As such, if the laboratory is equipped with a stereophotogrammetric device, this could be a relatively affordable, simple, and quick mean for the acquisition of the models. Moreover, for this study, the ventral surface of the bone was selected as the region of interest, whereas previous tests [12–15] considered the whole geometry of the bones. This may be among the shortcomings of the study. Further research may also consider the performance of the method on closed models of bones, where both anterior and posterior surface of the ilium are included in the model; this requires merging several models of the same object captured from different views. Even so, the analysis of the ventral iliac surface produced an effective screening test that identified all the correct matches for a preliminary sorting of innominate bones. Besides, previous studies that employed manual superimposition on humeri and temporal bones considered the mean and standard deviations of true pair-matches for calculating the threshold [12, 13]. Depending on the software, different values of the point-to-point distance may be available: Karell [27] states that the software Flexscan3D that was used for the pair-matching does not provide RMS distance values for the distance calculation. Conversely to Flexscan3D, the VAM software outputs the minimum, maximum, mean, standard deviation, and RMS values of the distance between the two models. More recently, the root of the mean distances of the points between surfaces has been used for the superimposition of clavicles [15], although only the automated version was applied. In this study, RMS point-to-point distance were used as a proxy to discriminate the matches and mismatches, which has been extensively applied to distance analysis of 3D models for personal identification, although when considering other structures, i.e., faces [28] and paranasal sinuses [20, 29–31]. The RMS distance is the root of the mean square of the point-to-point distances; this value provides a more thorough quantification of the similarity between models because it considers all the distances between two models, whereas the mean is the arithmetic average where the positive and negative values would elide each other [31].

The innominate bones used here presented different taphonomic profiles, as their anatomical completeness ranged from optimal to suboptimal. Previous studies on 3D pair-matching mostly included specimens that were anatomically complete or did not investigate the possible influence of taphonomy on the performance of the method. Only McWirther et al. [15] stated that the superimposition of fragmentary remains (artificially generated from CT scans) of clavicles yielded lower success rates, but only the automated superimposition performed by the proprietary software Viewbox 4 was assessed. This issue demands further attention, as anatomical completeness is an unlikely scenario for commingled skeletal remains which are recovered under various circumstances where taphonomy tends to influence the state of preservation, e.g., both recent and archaeological mass and secondary burials [3–7], flooded cemeteries [32, 33], or mass disasters [1, 34]. This study added a pilot investigation on the effects of taphonomy on the 3D superimposition of bone models. The results suggested that there is a possible relation between RMS distance of models and anatomical incompleteness of the skeletal remains, being the RMS distances values of the less preserved bones significantly higher than those of better-preserved remains. When considering bones with a suboptimal taphonomic profile, the point-to-point distance calculation generates a considerable number of false positive, which entails a decreased specificity rate. The performance may therefore be hampered by the state of preservation of the remains. However, this preliminary evidence is inferred based on initial results that should be further verified on larger samples, in order to provide sound evidence of the impact of taphonomy. Further research will focus on bones that present different states of preservation and will investigate whether the RMS distance values are influenced according to the taphonomic condition.

Eventually, especially in cases of highly commingled assemblages, anthropological re-association of commingled remains may not be decisive [35] and genetic profiling of the skeletal elements would be the ultimate test that provides the “smoking gun”, the conclusive evidence of a match or mismatch. However, extensive genetic testing on commingled bones may not be affordable for all countries or facilities [36]. Therefore, subscribing Karell et al. [12], 3D comparison of bilateral skeletal elements could help reducing the number of samples for DNA testing. On a final note, the 3D unmingling approaches introduce a powered tool that subjective methods lack, that is a numerical value indicating the match or mismatch. This is fundamental for strengthening the discipline of forensic anthropology, especially in the wake of judicial demands for more sound, verifiable and repeatable methods [37]. Moreover, the RMS distance values may support the results from osteometric analyses, which still depend upon the reference samples from which they are developed. New-generation pair-matching methods based on 3D approaches thus represent a valuable addition to be used together with the current “unmingling tool kit” [9]. The virtual techniques may therefore bolster the results of visual approaches based on morphological, taphonomic, pathological features and of osteometric methods for exclusion, as the gaps of one method  could be bridged by the information that are provided by another one. 

## Conclusions

This study tested a 3D method to sort innominate bones. For the first time, this approach included digital models of the ventral iliac surface that were acquired with a stereophotogrammetric device, showing that this acquisition mean provides accurate models that can be used for the 3D pair-matching. A low RMS distance value generally indicates a true pair-match of innominate bones that should be further verified, because of the possible presence of false positives. For this reason, at this stage, the method serves as a screening test that provides a numerical value for the verification of the match to be used in conjunction with other methods.

The preliminary results introduce the possible relation between the state of preservation of the remains and the outcome of the point-to-point distance calculation. This study provides therefore only pilot evidence on the influence of taphonomy on the results of 3D-3D superimposition of bone models, which would benefit from further research. Eventually, this paper contributes to expand the new-generation digital pair-matching methods providing an addition to the kit that may complete the results of traditional techniques.

## Data Availability

All data analysed during this study are included in this published article.
